# The Role of Antioxidant Minerals in the Pathophysiology and Treatment of Endometriosis—Systematic Review

**DOI:** 10.3390/antiox14101238

**Published:** 2025-10-15

**Authors:** Kamila Pokorska-Niewiada, Maciej Ziętek, Iwona Szydłowska, Karina Ryterska, Małgorzata Szczuko

**Affiliations:** 1 Department of Toxicology, Dairy Technology and Food Storage, West Pomeranian University of Technology in Szczecin, 71-454 Szczecin, Poland; 2 Department of Perinatology, Obstetrics and Gynecology, Pomeranian Medical University in Szczecin, 71-460 Szczecin, Poland; maciej.zietek@pum.edu.pl; 3 Department of General Pharmacology and Pharmacoeconomics, Pomeranian Medical University in Szczecin, 71-460 Szczecin, Poland; 4 Department of Gynecology, Endocrinology and Gynecological Oncology, Pomeranian Medical University in Szczecin, 71-460 Szczecin, Poland; iwona.szydlowska@pum.edu.pl; 5 Department of Human Nutrition and Metabolomics, Pomeranian Medical University in Szczecin, 71-460 Szczecin, Poland; 6 Department of Bromatology and Nutritional Diagnostics, Pomeranian Medical University in Szczecin, 71-460 Szczecin, Poland

**Keywords:** antioxidants, oxidative stress, endometriosis, elements

## Abstract

Endometriosis (EM) is a chronic gynecological disease of women of reproductive age. Due to the lack of a known cause, treatment is limited to reducing symptoms associated with pelvic pain and infertility. The aim was to determine the contribution of minerals and their supplementation to the EM. This systematic review was conducted in accordance with the PRISMA guidelines. The literature was searched in four databases: PubMed, Scopus, Web of Science and Google Scholar for the keywords ‘oxidative stress’, ‘supplementation’, ‘trace elements’, ‘metalloestrogens’, ‘antioxidants’, ‘zinc’, ‘copper’, ‘manganese’, “selenium”, ‘Zn/Cu’ and ‘molybdenum’ published by the end of June 2025. It turns out that there are significant differences in the biochemical analysis of elements between women with EM and healthy women. Most studies showed decreased zinc levels in women with EM, and copper and molybdenum were usually in comparable amounts, whereas the results for selenium are inconclusive. The lack of direct clinical trials of antioxidant element supplementation, coupled with evidence of differences in their levels between women with EM and healthy women, warrants further, more detailed analysis. Studies should be expanded to include dose–response analyses and potential threshold effects. This will allow for the assessment of the clinical usefulness of supplementation or dietary enrichment as an adjunctive therapeutic approach in the treatment of EM symptoms.

## 1. Introduction

Endometriosis (EM) is one of the most common chronic gynecological diseases in women of reproductive age. It affects approximately 10–15% of women worldwide [1,2]. The likelihood of developing EM increases with painful menstruation (40–60%), uterine pain (71–87%) and infertility (21–47%) [3,4]. The origin of this disease is ectopic implantation of endometrial-like tissue outside the uterine cavity (ovaries, uterosacral ligaments, peritoneum, rectovaginal pouch, vagina, intestines, bladder), leading to chronic inflammation [5,6]. EM manifests itself as severe pain during menstruation and sexual activity, bowel movements and/or urination, chronic pelvic pain, abdominal bloating, nausea, fatigue, and sometimes depression, anxiety and infertility [7]. Unfortunately, treatment is limited to alleviating symptoms. To date, there is no effective therapy that allows for complete recovery. Patients are offered painkillers, hormone therapy, surgical removal of lesions and non-pharmacological treatments. Hormonal treatment of EM aims to block the hypothalamic-pituitary-ovarian axis or induce pseudodecidualization of the endometrium. This leads to the cessation of menstruation and thus prevents endometrial cells from implanting outside the uterine cavity and the development of EM.

Gonadotropin-Releasing Hormone Agonists (leuprolide, goserelin, nafarelin) and antagonists (abarelix, cetrorelix, degarelix, ganirelix, elagolix, relugolix) are effective in treating EM because they affect the functioning of the pituitary gland and ovaries.

Progestogens are mainly used for long-term treatment (dienogest, norethindrone acetate, medroxyprogesterone acetate) and act in many places. Bi-component oral contraceptives are also used to relieve the symptoms of EM by inhibiting ovarian function [8].

Despite many years of research on EM, its etiology and pathogenesis, it is still considered a poorly understood disease [8,9,10].

Factors that may increase the likelihood of developing EM include early onset of menstruation (before the age of 11), short menstrual cycles (less than 27 days), low BMI, reproductive organ malformations and alcohol abuse. The influence of environmental factors on the development and progression of this disease is also considered. A factor that receives particular attention is tobacco smoking [11,12,13]. The biological mechanisms linking smoking to EM remain complex and not fully understood. Epidemiological data suggest that smoking is not only a risk factor for EM, but may also exacerbate its course [12,14]. The results are sometimes contradictory, as in the case of Nouri et al. [12], who showed that cigarette smoking reduces the risk of EM.

Due to the lack of an effective cure for EM, other solutions are being sought to alleviate the symptoms associated with this disease. There are reports indicating that a proper diet and a healthy lifestyle can play a significant role in alleviating symptoms and improving quality of life. This is confirmed by the results of numerous observational and clinical studies [15,16,17,18,19,20]. The introduction of a Mediterranean, Ketogenic, low-FODMAP or gluten-free diet to the nutrition of women with EM significantly reduced the symptoms of the disease, including pain [21,22,23]. This is because these diets contain high amounts of antioxidants, which can alleviate inflammation and reduce oxidative stress [24,25,26]. However, consuming large amounts of red and processed meat, trans fats, and refined sugar increases the risk of EM and exacerbates its symptoms [27]. Although there is evidence of the beneficial effect of antioxidants on the course of EM and their supplementation is recommended, there are very few reports on one of the groups of antioxidants, which include some elements. Elements create many enzymes, react with vitamins and influence the proper course of many metabolic processes. Their excess or deficiency may indicate developing disease, so it is crucial to examine the composition of a woman’s blood, urine, and follicular fluid. There are reports suggesting such differences [15,28,29,30,31,32,33]. The aim of this review was to collect and summarize previous reports on differences in mineral composition in women with EM compared to women without this disease. In addition, attention was paid to the relationships between elements that may strengthen or weaken their effects. Unfortunately, there is currently no research on the introduction of supplementation solely with a given element with antioxidant properties.

## 2. Materials and Methods

To ensure effective methodological integrity, validity and quality, the PRISMA database was used [34]. This review primarily used the Research Gate databases, Scopus-indexed articles, Google Scholar, Science Direct, and PubMed. Each database search was carried out independently, and the selected articles were juxtaposed together. The following keywords were used in the search process: ‘endometriosis’ in combination with the terms: ‘oxidative stress’, ‘supplementation’, ‘trace elements’, ‘elements’, ‘microelements, ‘metalloestrogens’, ‘antioxidants’, ‘zinc’, ‘copper’, ‘manganese’, “selenium”, ‘Zn/Cu’, ‘molybdenum’. Relevant articles and review papers on research into the relationship between EM and selected elements were included, exclusively articles written in English and Polish. Similar articles, as suggested by the databases, were also searched for inclusion. In addition, references to all relevant publications and systematic reviews identified during the initial search were also searched. Reference lists of selected articles were also searched manually to identify any additional related documents, and relevant publications were consulted with experts in the field.

The inclusion criteria were as follows: original research, results of biochemical analyses, control group, English or Polish language, articles from peer-reviewed scientific journals. The exclusion criteria were as follows: animal studies, retracted articles, articles without free access, incomplete/insufficient data.

**Figure 1:**
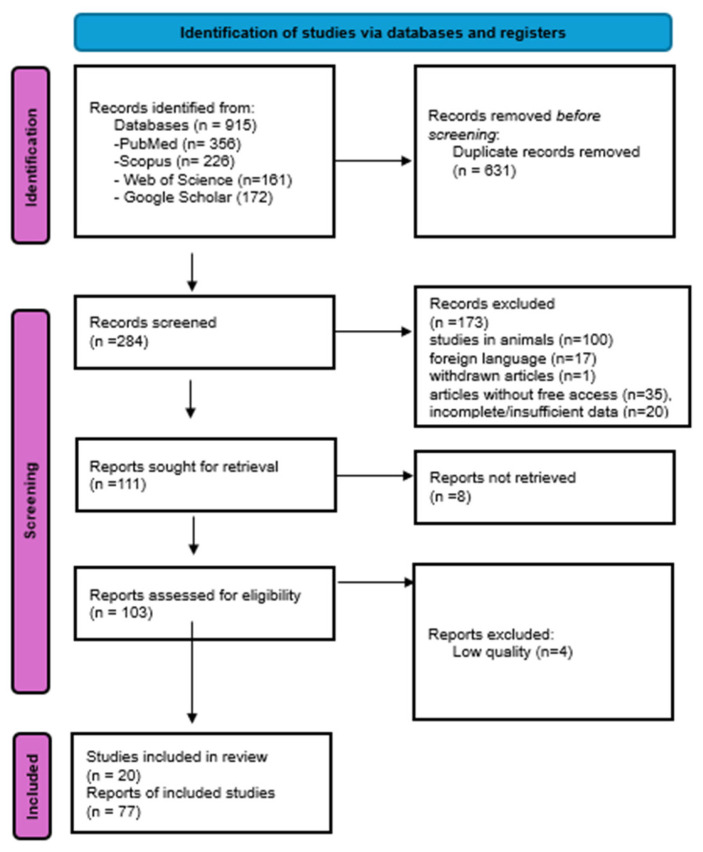
PRISMA Flow diagram.

This systematic review was conducted in accordance with the PRISMA guidelines [Supplementary Materials]. Two authors independently searched the literature and, after eliminating duplicate articles, assessed their eligibility according to the above criteria. In addition, they extracted data from each article included in the review. These data included, among others, the title, author, journal, year of publication, study population, results, comparisons, and outcomes. Two authors confirmed this final list of topics, which includes the titles presented in the Section 3 of this review. To select the preferred articles for this study, a thorough review of all articles was conducted by reading their titles and abstracts. To ensure rigorous methodology and reduce bias, the article selection process for this study was multi-step (Figure 1). By using this approach, all data that were relevant, of good quality, and aligned with the study objectives were included in the study.

## 3. Results

### 3.1. The Mechanism of Endometriosis Development

In the search for an explanation for the development of EM, attention is increasingly being drawn to the role of oxidative stress. It is perceived as an imbalance between reactive oxygen species (ROS) and antioxidants, which may cause a general inflammatory reaction in the peritoneal cavity [4,16]. ROS accumulation leads to activation of the most important cell proliferation signaling pathways (Raf/MEK/ERK and mTOR). The activation of these pathways enables the survival and proliferation of ectopic endometrial cells, causing the progression of EM [16,17,18].

The importance of oxidative stress in the development of EM has already been analyzed by many scientists [4,16,17,18,19,20]. It has been shown, among others, that women with EM may have elevated oxidative stress parameters, with a significant decrease in plasma superoxide dismutase 1 (SOD1) levels and an increase in lipid peroxidation enzymes [20,21,22].

Excess ROS causes, among others, the following:

- Increased production of pro-inflammatory cytokines: IL-6 (interleukin 6), TNF-α (tumor necrosis factor alpha), IL-1β (interleukin 1 beta) [16];
- Stimulation of VEGF (vascular endothelial growth factor) production, which stimulates angiogenesis [22];
- Stimulation of MAPK (mitogen-activated protein kinase) pathways [23];
- Activation of Notch pathways [24];
- Excessive formation of connective tissue (fibrosis)—adhesions and scars [18];
- Stimulation of adhesion processes [25];
- Activation of the transcription factor NF-κB (nuclear factor kappa-B) [16].

In recent years, increasing attention has been put on the relationship between trace element homeostasis disorders and the risk of EM development [21,26].

In search of an explanation for the causes of EM, scientists compared the results of biochemical analyses of patients affected by EM with those of patients without EM (but with problems conceiving a child). These included studies of trace element content in whole blood, follicular fluid, serum and urine [Table 1].

It turns out that there are significant differences in the biochemical analyses of elements between women with EM and healthy women in this respect, which confirms the hypothesis of an important role in the course of EM [15,30]. However, studies on the relationship between EM and the amount of essential trace elements (such as Zn, Se, Cu, Co and Mo) are very limited and difficult to interpret due to high variability.

### 3.2. Minerals and Their Antioxidant Effects

#### 3.2.1. Selenium (Se)

Selenium is an element that is part of two key amino acids: selenomethionine and selenocysteine. These amino acids build enzymes essential for the proper functioning of the body, acting primarily as antioxidant defense mechanisms:

- Glutathione peroxidases (GPx1, GPx2, GPx3, GPx4, GPx6)—antioxidant properties—reduction of hydrogen peroxide, organic peroxides, including phospholipid peroxides
- Thioredoxin reductases (TrxR1, TrxR2, RGR)—antioxidant properties—reduction of thioredoxins and protein disulphides, participation in DNA synthesis and apoptosis processes
- Iodothyronine deiodinases (DIO1, DIO2, DIO3)—activate/deactivate thyroid hormones
- Selenoproteins (SelenoW, SelenoH, SelenoT, SelenoP, SelenoM, SelenoN, SelenoK, SelenoS, SelenoV, SelenoI, SelenoO)—control of redox potential in the cell, protection of neurons against oxidative stress [6,35,36,37].

Patients with EM often have increased levels of oxidative stress and decreased levels of antioxidants, including selenium [6,38,39]. Studies have shown that the glutathione peroxidase (GPX) system, which contains selenium, is one of the major sulfhydryl-dependent antioxidant systems that combat oxidative stress [40]. The GPX system is widely distributed in human epithelial cells, including the glandular epithelium of the uterine endometrium. By participating in the elimination of hydroxyl radicals, it effectively reduces oxidative stress and protects the endometrium [6,24,38,41].

#### 3.2.2. Zinc (Zn)

Zn acts as a cofactor for over 300 enzymes involved in, among other things, protein synthesis, proper functioning of the immune system, cell division and differentiation, and tissue regeneration [30]. Zinc has strong antioxidant properties (it is a component of copper-zinc superoxide dismutase (Cu/Zn-SOD) (SOD1)) and anti-inflammatory properties, which are essential for maintaining cellular redox homeostasis.

Zinc supports key reproductive processes by facilitating DNA synthesis, cell division and gene expression [15,42,43]. Although no direct link between Cu/Zn-SOD and EM has been demonstrated, oxidative stress influences the development of EM.

Furthermore, a significant reduction in SOD1 and an increase in lipid peroxidase were observed in the plasma of women with EM [44,45].

In the context of EM, zinc

- Plays an important role in hormonal changes and may support the regulation of estrogen levels [3,32],
- Modulates immune responses and reduces oxidative stress [30,32,43],
- Reduces the level of matrix metalloproteinases (MMPs), especially MMP-2 and MMP-9, reducing the invasiveness of endometrial lesions [6,46],
- Is an important component of ZEB1 and ZEB2 molecules, which are involved in the epithelial–mesenchymal transition (EMT) process in EM and are associated with the severity of the disease. However, it has not been proven that zinc deficiency disrupts the expression of ZEB1 and ZEB2 [6].

The results of clinical studies are not uniform [Table 1]. Contradictory results may be due, among others, to differences in lifestyle and research methodology, including the method of sample collection, preparation and storage [15,47].

#### 3.2.3. Copper (Cu)

Copper (Cu) primarily acts as a cofactor for enzymes and a structural element of over 30 proteins involved in charge transfer processes.

The most important enzymes include

- Cu Zn superoxide dismutase (Cu Zn-SOD or SOD 1), which prevents damage to major biomolecules such as DNA, lipids and proteins by catalyzing the reduction of superoxide radicals to hydrogen peroxide [15].
- Cytochrome C oxidase (COX)—is an electron acceptor in the mitochondrial respiratory chain [48].
- Tyrosinase—essential for melanin production, but has the ability to generate free radicals in the process of catalyzing the reaction. This can stimulate the formation of oxidative stress [49,50].
- Catechol oxidase is an enzyme involved in the metabolism of catecholamines (e.g., adrenaline, dopamine). However, during this process, free radicals may be produced, which may contribute to oxidative stress [51].

Copper regarding EM:

- It is a component of amino oxidase 3, an enzyme present in endometrial lesions [28,52].
- It can interact with estrogen receptors, influencing the development and functioning of endometrial cells [15].
- Helps protect cells against damage caused by free radicals—presence in SOD [30].
- Copper stimulates the production of reactive oxygen species, which may cause lipid peroxidation [30,53].

Higher serum Cu concentrations have been reported in women with EM compared to healthy women [29,31,33,53,54]. Table 1 summarizes Cu concentrations in various biological matrices of women with EM compared with controls. However, due to the limited sample size in these studies, elucidating the specific mechanism of disease development is quite difficult.

#### 3.2.4. Manganese (Mn)

Manganese (Mn) is an element involved in glucose and lipid metabolism, stimulating the synthesis of proteins and vitamins (C and B), regulating the hormonal system and neuronal activity, and catalyzing hematopoiesis.

It is a cofactor of many enzymes (including arginase, glutamine synthetase, phosphoenolpyruvate decarboxylase, and manganese superoxide dismutase (MnSOD)) that reduce oxidative stress induced by free radicals [55].

Lower manganese superoxide dismutase activity was found in patients with EM compared to the control group [56,57]. However, many studies on EM have not observed statistically significant differences [Table 1]. Therefore, it seems that despite the presence of manganese in SOD, this element does not play a significant role in the development of EM [19,57]. However, further studies are needed to confirm or refute this hypothesis.

#### 3.2.5. Molybdenum (Mo)

Molybdenum is an essential trace element that is toxic in high doses. In humans, molybdenum is a component of four enzymes:

- Sulfite oxidase (SUOX) found in mitochondria. It catalyzes the conversion of sulfite to sulfate and participates in the reduction of nitrates (III) to nitrogen oxide.
- Xanthine oxidase (XOR), which is involved in the breakdown of purines and the production of reactive oxygen species (ROS).
- Aldehyde oxidase (AOX), found primarily in the liver. It is involved in detoxification. particularly in the metabolism of alcohol and other compounds, and is important in drug metabolism.
- Mitochondrial amidoxime reducing component (mARC)—its exact function is not yet known [58,59].

Molybdenum in EM:

In EM, xanthine oxidase expression is often higher than normal, especially in ectopic endometrium (tissue located outside the uterus). This increased expression contributes to increased ROS production, leading to oxidative stress [58,60,61].

Few studies have been conducted on the use of this element in the diagnosis of EM. Su et al. [15] indicated that Mo concentration in patients’ blood showed a negative correlation with the risk of EM, whereas in follicular fluid it showed a positive correlation. Due to these contradictory results, it was not possible to draw any conclusions regarding Mo concentration and its association with the risk of EM.

### 3.3. Interactions Between Minerals

The problem of the presence of elements in the body concerns not only their concentrations, but also the interactions between them (especially antagonistic relationships), which are extremely important.

#### 3.3.1. Cu/Zn

One of the most important aspects of EM development is the copper to zinc (Cu/Zn) ratio. This is an important diagnostic factor because it indicates physiological balance or redox imbalances that may lead to pathological conditions associated with oxidative stress [30,62,63].

An increased Cu/Zn ratio influences antioxidant defense and immune functions, which may favor the development of EM [30]. Many researchers confirm this assumption [15,29,30]. However, there are studies indicating the lack of such relationships [64] or even the existence of positive correlations between these elements [e.g., in follicular fluid [15].

The differences in the relationship between blood and follicular fluid may be due to the presence of the blood–follicle barrier. This may indicate that this barrier operates by a more complex mechanism than the previously assumed physical filtration [65,66].

#### 3.3.2. Se-Pb, Se-Cd, Se-As, Se-Hg

The antagonism of selenium towards harmful elements (lead, cadmium, arsenic and mercury) enables their intoxication by forming inactive and non-toxic complexes with them [67,68].

#### 3.3.3. Mo-Cu, Mo-Fe

The antagonistic effect of molybdenum on copper and iron has been observed [15,51].

#### 3.3.4. Mo-W, Mo-Pb, Mo-Na

Tungsten, lead and sodium are considered to act as antagonists of molybdenum and cause its deficiency in the body [31,69,70].

#### 3.3.5. Mn-Fe

Manganese toxicity may increase iron deficiency. Manganese competes with iron for absorption and binding sites, as well as for transport proteins [71,72,73].

## 4. Potential Clinical Applications

The treatment of EM is a complex process. It primarily involves

- Pharmacotherapy, mainly non-steroidal anti-inflammatory drugs (NSAIDs) and hormonal drugs [74].
- Surgical treatment.

However, these methods are not entirely effective, and both have adverse effects. Taking medication is associated with side effects such as nausea, vomiting, gastrointestinal reactions, liver and kidney dysfunction, stomach ulcers and thrombosis. On the other hand, surgical removal of EM lesions does not always solve the problem. The disease often recurs, in up to 50% of cases within 5 years [75].

Therefore, alternative methods of treating EM are being sought, such as dietary and lifestyle modifications.

### Supplementation as a Treatment Method

Analyzing individual elements with antioxidant properties and their significance in the development and course of EM, it seems that supplementation with these elements will help solve the problem of EM. To date, there are reports in the literature that antioxidant supplementation has reduced painful menstruation and oxidative stress [76,77,78,79,80]. However, there is insufficient research on this topic. This may be due to the issue of interactions between elements (mentioned earlier), as well as the risk of side effects associated with excessive intake of selected antioxidants [15].

When considering selenium supplementation, great caution should be exercised, as chronic exposure to high doses of this element may adversely affect reproduction (negative correlation between EM and glutathione peroxidase) [15,81]. There are no clinical studies on the possibility of using selenium as a treatment for EM.

Supplementation may be justified by the fact that the human body cannot store zinc, and the only way to maintain adequate levels of this element is to obtain it from the daily diet [3]. When attempting to introduce zinc supplementation, caution should be exercised as it may adversely affect the immune system, iron metabolism, copper homeostasis and lead to a decrease in HDL cholesterol concentration. Zinc is involved in enzymatic activity, immune modulation, and interactions with MMP [3,15,30,82].

There are only isolated research results confirming the clinical efficacy of zinc and other antioxidant supplementation in EM [15,78,83,84].

Introducing excessive amounts of copper into the body can cause negative effects. High Cu levels mediate ROS formation and ROS-induced damage [15]. Excessive amounts of copper accumulate in the liver, brain and cornea of the eye, resulting in damage to these organs. Copper accumulation in the liver may contribute to the development of liver cancer. Excess copper can negatively affect cognitive abilities.

Under physiological conditions, manganese and iron compete for transferrin binding. In iron deficiency anemia, manganese absorption is also impaired due to competition for transport proteins. Vitamins B1, E, phosphorus, and calcium (in moderate amounts) facilitate manganese absorption in the gastrointestinal tract, whereas excessive calcium and phosphorus intake impairs it [6].

Copper sulfate increases the excretion of molybdenum from the blood. Ammonium thiomolybdate (a soluble molybdenum salt) is a copper antagonist and impairs its utilization in the body. Copper and iron deficiency increases molybdenum levels in the body [8].

## 5. Elements with Antioxidant Properties as Part of Endometriosis Treatment

Many researchers have pointed out a potential link between the consumption of certain foods and the risk of developing EM. There are numerous reports highlighting the impact of nutrients on chronic inflammation and excessive oxidative stress, which occur in EM [85,86,87,88]. Studies indicate that women with EM have a lower intake of antioxidants in their diet, including zinc. Introducing antioxidant supplementation can reduce painful periods and oxidative stress [30,78,89]. Adopting a Mediterranean diet is an example of improving the quality of life (reducing pain) of patients with EM [86,90,91].

In the diet, selenium is found in cereals, vegetables (especially garlic, mushrooms, dry legume seeds), fish, seafood, meat, offal (especially kidneys), dairy products and nuts The bioavailability of selenium is high—over 90%. Selenium is best absorbed from plant-based products (e.g., wheat, corn), but much less so from certain fish (e.g., tuna) [67,92].

The main sources of zinc in the human diet are meat and meat products, cereals and cereal products, and milk and dairy products [4]. The absorption of zinc from the diet is 20–40%, with this element being better absorbed from animal products than from plant products [67]. The absorption of zinc is limited by the presence of copper, non-heme iron and calcium. The amounts of zinc typically found in food do not lead to excessive intake [82].

Foods rich in copper include liver, wheat germ and bran, oatmeal, offal (especially liver), nuts, cocoa and sunflower seeds. Copper absorption from an average diet is 35–50%. Copper is better absorbed from a diet rich in animal protein than from a diet containing mainly plant proteins. Sulfides, phytates, sucrose, fructose, sulfur-containing amino acids, calcium, phosphorus, zinc and iron, among others, have a negative effect on copper absorption. A typical diet does not pose a risk of excessive copper intake [93].

Foods rich in manganese include whole-grain bread, dry legume seeds, e.g., beans, peas, buckwheat, nuts and tea. Excess manganese tends to accumulate in the liver, pancreas, bones, kidneys and especially in the brain, which is the main site of its toxic effects [94,95]. Since manganese is commonly found in food in sufficiently high concentrations, its deficiency does not pose a threat to public health [55].

Molybdenum occurs in food in trace amounts in the form of soluble molybdates. Foods rich in molybdenum include legumes (especially beans), whole grain products, offal (liver, kidneys), nuts, vegetables (including asparagus and some cruciferous vegetables), and milk. The absorption of molybdenum from the diet in adults ranges from 40% to 100%. Typically, dietary intake of molybdenum significantly exceeds the requirement for this nutrient [96,97].

## 6. Conclusions and Directions for Future Research

EM is a complex, chronic gynecological disease characterized by a multifactorial etiology, including genetic, environmental, immunological, and inflammatory factors [6]. Treatment only alleviates symptoms, but there is currently no cure. Therefore, it is important to clarify possible differences in biochemical markers between samples collected from affected and healthy women.

Given the ubiquity of antioxidants in the body and the multitude of reactions they mediate, it is reasonable to assume that EM is associated with differences in their levels. Elevated/decreased levels of these elements may be involved in the pathogenesis of EM. Further multicenter prospective studies with a larger number of individuals are needed, as well as studies involving additional tissues such as follicular fluid, urine, and endometrial tissue. Parameters, including markers associated with oxidative stress, may also be required [15,30]. The lack of direct clinical trials of antioxidant element supplementation, coupled with evidence of differences in their levels between women with EM and healthy women, warrants further, more detailed analysis. Studies should be expanded to include dose–response analyses and potential threshold effects. This will allow for the assessment of the clinical usefulness of supplementation or dietary enrichment as an adjunctive therapeutic approach in the treatment of EM symptoms [30,84].

In addition, research is needed to investigate the mechanisms by which specific nutrients modulate inflammation and hormonal balance, as well as the relationship between diet, gut microbiota and EM. It appears that a low-protein, and therefore vegetarian, diet may not be appropriate for women with EM due to reduced absorption of some minerals [30,86].

One of the most valuable contributions of this review to the medical community, particularly gynecologists, is that every patient with EM should undergo tests that include the determination of minerals such as zinc, selenium, manganese, molybdenum and copper and verify that their levels are within the biological reference intervals; otherwise, they should be referred to a nutritionist to design a special diet according to their body mass index, which in this way can contribute as an alternative for the treatment of this gynecological condition that affects many women worldwide.

Strengths of the review:

This review addresses EM from the perspective of a diet rich in antioxidants and a healthy lifestyle, which could reduce the risk of complications from this condition. A thorough search was carried out in the databases where articles published until June 2025 were considered. Articles are shown in which scientists compared the results of biochemical analyses of patients with EM with those of patients without EM. The parameters analyzed included studies of trace element content in whole blood, follicular fluid, serum, and urine. Not only was an analysis of selected elements with antioxidant activity carried out, but also, taking into account the relationships between them, an analysis of interactions between minerals was carried out.

Standard EM treatments are associated with side effects and do not guarantee a cure. Therefore, in this review, we recommend a healthy lifestyle and a balanced diet. These recommendations are important for everyone, but for women with EM, it is especially important, as taking antioxidant supplements can reduce menstrual pain and oxidative stress.

One of the most valuable contributions of this review is the need for detailed testing of patients with EM. The test package should be expanded to include elements (including zinc, selenium, manganese, molybdenum and copper) and others, and it should be checked whether the patients’ levels are within biological reference ranges. If any deviations from the norm are detected, the patient should be referred to a dietitian who will develop a personalized diet. This approach can complement conventional treatment and, in some cases, may be an alternative to medications and surgery.

Weakness:

Research on the relationship between EM and the amount of essential trace elements (such as Zn, Se, Cu, Co, and Mo) is very limited and difficult to interpret due to high variability.

## Figures and Tables

**Table 1:** Comparison of mineral content in the analyzed groups.

Element	Body Fluids	Number of Samples	Women with Endometriosis	Control Group	Observations	References
Se	blood	217 cases/234 controls	151.15 μg/L	131.63 μg/L	Significantly increased Sn levels in EM group	[15]
Se	serum	302 cases/ 302 controls	94.71 μg/L	97.71 μg/L	Significantly decreased Se levels in EM group	[29]
Se	follicular fluid	182 cases/203 controls	52.65 μg/L	40.51 μg/L	Significantly increased Se levels in EM group	[15]
Zn	blood	68 cases/122 controls	6.72 mg/L	11.86 mg/L	Significantly decreased Zn levels in EM group	[28]
Zn	serum	302 cases/302 controls	921.68 μg/L	945.98 μg/L	Significantly decreased Zn levels in EM group	[29]
Zn	serum	42 cases/44 controls	1.01 ± 59.2 μg/L	1.29 ± 62.22 μg/L	Significantly decreased Zn levels in EM group	[32]
Zn	blood	217 cases/234 controls	8666.4 μg/L	4847.88 μg/L	Significantly increased Zn levels in EM group	[15]
Zn	serum	568 cases/819 controls	14.6 μmol/L	15.1 μmol/L	Significantly decreased Zn levels in EM group	[30]
Zn	follicular fluid	182 cases/203 controls	475.91 μg/L	290.11 μg/L	Significantly increased Zn levels in EM group	[15]
Zn	urine	190 cases/283 controls	265.64 μg/L	283.96 μg/L	No significant differences	[31]
Cu	serum	302 cases/302 controls	1528.25 μg/L	1465.56 μg/L	Significantly increased Cu levels in EM group	[29]
Cu	serum	31 cases/41 controls	1088 ± 273.58 μg/mL	811.20 ± 265.77 μg/mL	Significantly increased Cu levels in EM group	[33]
Cu	blood	68 cases/122 controls	0.39 mg/L	0.48 mg/L	No significant differences	[28]
Cu	serum	568 cases/819 controls	15.77 μmol/L	15.6 μmol/L	No significant differences	[30]
Cu	blood	217 cases/234 controls	963.05 μg/L	991.18 μg/L	No significant differences	[15]
Cu	follicular fluid	182 cases/203 controls	759.43 μg/L	479.51 μg/L	Significantly increased Cu levels in EM group	[15]
Cu	urine	190 cases/283 controls	10.64 μg/L	10.33 μg/L	No significant differences	[31]
Mo	blood	217 cases/234 controls	1.19 μg/L	1.37 μg/L	No significant differences	[15]
Mo	follicular fluid	182 cases/203 controls	0.99 μg/L	0.56 μg/L	Significantly increased Mo levels in EM group	[15]
Mo	serum	302 cases/302 controls	1.23 μg/L	1.13 μg/L	Significantly increased Mo levels in EM group	[29]
Mo	urine	190 cases/283 controls	44.49 μg/L	44.56 μg/L	No significant differences	[31]
Mn	urine	190 cases/283 controls	1.41 μg/L	1.39 μg/L	No significant differences	[31]
Mn	blood	68 cases/122 controls	0.72 μg/L	0.65 μg/L	No significant differences	[28]

## Data Availability

Dataset available on request from the authors.

## Supplementary Materials

The following supporting information can be downloaded at https://www.mdpi.com/article/10.3390/antiox14101238/s1.

## Abbreviations

The following abbreviations are used in this manuscript:

BMI	Body Mass Index
EM	Endometriosis
GPX	glutathione peroxidase
MAPK	mitogen-activated protein kinase
MMP	metalloproteinases
ROS	reactive oxygen species
